# Stimulant medication and symptom interrelations in children, adolescents and adults with attention-deficit/hyperactivity disorder

**DOI:** 10.1007/s00787-024-02610-8

**Published:** 2024-11-11

**Authors:** Zarah van der Pal, Hilde M. Geurts, Jonas M. B. Haslbeck, Alex van Keeken, Anne Marijn Bruijn, Linda Douw, Daan van Rooij, Barbara Franke, Jan Buitelaar, Nanda Lambregts-Rommelse, Catharina Hartman, Jaap Oosterlaan, Marjolein Luman, Liesbeth Reneman, Pieter J. Hoekstra, Tessa F. Blanken, Anouk Schrantee

**Affiliations:** 1 Department of Radiology & Nuclear Medicine, Amsterdam University Medical Center Location University of Amsterdam, Amsterdam, The Netherlands; 2 Division of Brain & Cognition, Department of Psychology, University of Amsterdam, Amsterdam, The Netherlands; 3 Department of Psychological Methods, University of Amsterdam, Amsterdam, The Netherlands; 4 Department of Clinical Psychological Science, Maastricht University, Maastricht, The Netherlands; 5 Department of Anatomy & Neurosciences, Amsterdam University Medical Center Location Vrije Universiteit Amsterdam, Amsterdam, The Netherlands; 6 Donders Institute for Brain, Cognition and Behavior, Donders Centre for Cognitive Neuroimaging, Department of Cognitive Neuroscience, Radboud University Medical Center, Nijmegen, The Netherlands; 7 Department of Human Genetics, Radboud University Medical Center, Nijmegen, The Netherlands; 8 Department of Psychiatry, University Medical Center Groningen, Groningen, The Netherlands; 9 Department of Clinical Neuropsychology, Vrije Universiteit, Amsterdam, The Netherlands; 10 Department of Child and Adolescent Psychiatry, University of Groningen-University Medical Center Groningen, Groningen, The Netherlands

**Keywords:** Attention-deficit/hyperactivity disorder, Stimulant medication, Symptom networks, Psychopharmacology

## Abstract

Stimulant medication is effective in alleviating overall symptom severity of attention-deficit/hyperactivity disorder (ADHD), yet interindividual variability in treatment response and tolerability still exists. While network analysis has identified differences in ADHD symptom relations, the impact of stimulant medication remains unexplored. Increased understanding of this association could provide valuable insights for optimizing treatment approaches for individuals with ADHD. In this study, we compared and characterized ADHD symptom networks (including 18 ADHD symptoms) between stimulant-treated (*n* = 348) and untreated (*n* = 70) individuals with ADHD and non-ADHD controls (NACs; *n* = 444). Moreover, we compared symptom networks between subgroups defined by their stimulant treatment trajectory (early-and-intense use, late-and-moderate use). Stimulant-treated individuals with ADHD showed stronger associations between symptoms, compared with untreated individuals with ADHD and NACs. We found no differences in symptom networks between the stimulant treatment trajectory subgroups. Prospective longitudinal studies are needed to disentangle whether the identified differences stem from treatment or pre-existing factors.

**Supplementary Information:**

The online version contains supplementary material available at 10.1007/s00787-024-02610-8.

## Introduction

Attention-deficit/hyperactivity disorder (ADHD) is a heterogeneous neurodevelopmental disorder^1^, characterized by age-inappropriate levels of inattention, hyperactivity and/or impulsivity that interfere with typical development or functioning [3–5]. Stimulant medication, such as methylphenidate- and dexamphetamine-based formulations, is commonly prescribed to children, adolescents, and adults with ADHD [6, 7], and has been widely reported to alleviate overall ADHD symptom severity [8–12]. While this approach provides valuable information about the global burden of ADHD symptoms and the efficacy of stimulant medication in reducing overall symptom severity, it may overlook important nuances in the underlying dynamics of the disorder. The network theory of psychopathology offers a promising framework to address this limitation by shifting the focus from symptom counts to exploring the intricate interactions and dependencies between individual symptoms [13]. Network analysis may be used to gain deeper understanding of the unique associations between symptoms, and which (clusters of) symptoms may potentially be targeted in treatment of ADHD. Interestingly, thus far medication use has not been taken into account when investigating the relations between individual symptoms.

Studies using symptom network analysis have reported developmental patterns and specific symptoms that play an important role in ADHD symptom networks. For instance, symptom network analysis in children with ADHD revealed that hyperactive-impulsive symptoms had greater relative importance compared with inattentive symptoms [14]. In a different study, both hyperactive-impulsive and inattentive symptoms were identified as central to symptom networks across three time points in a longitudinal sample of children and young adolescents with ADHD [15]. Symptoms that are central to the network are strongly connected to other symptoms and may influence, or be influenced by, the expression of these other symptoms. It has therefore been suggested that central symptoms have potential to predict clinical outcome [15], and may be viable treatment targets in ADHD [16].

However, the effects of treatment itself on symptom network properties in ADHD remain unexplored. It is essential to increase our understanding of how the relations between symptoms differ between individuals with ADHD that use stimulant medication, compared with those that do not, as this may provide new leads for optimizing treatment approaches and improving outcomes for individuals with ADHD.

Therefore, the present study aimed to investigate how exposure to stimulant medication relates to ADHD symptom networks, evaluating the strength of associations and (local) network structure in stimulant-treated and untreated individuals with ADHD, as well as non-ADHD controls (NACs). We expected that effective treatment will lead to alterations in the relations between symptoms, potentially disrupting pathological connections and establishing healthier patterns. Thus, we hypothesized that exposure to stimulant treatment would be associated with a symptom network that is more similar to the network in NACs, compared with untreated individuals with ADHD.

## Methods

### Participants and measurements

#### Sample

The data used in this study were collected as part of the prospective NeuroIMAGE project [17], a Dutch follow-up study of the International Multicenter ADHD Genetics (IMAGE) project [18–20]. The first wave of this two-site follow-up study (Radboud University Medical Centre, Nijmegen; Vrije Universiteit Amsterdam), conducted between 2009 and 2012, aimed to identify genetic, neurobiological and environmental causes underlying ADHD and its course during development. The total NeuroIMAGE sample consisted of 1085 Caucasian participants aged 7–29 years old. Written informed consent was obtained from all participants aged 12 years and older. Parents also provided written informed consent for participants under 18. The NeuroIMAGE study was approved by the medical ethical committees of the VU University Medical Centre and Radboud University Medical Centre (NL23894.091.08 [17]).

ADHD diagnosis was established based on outcomes of a semi-structured interview with parents (Kiddie – Schedule for Affective Disorders and Schizophrenia Present and Lifetime Version, K-SADS-PL [21]), complemented by scores on Conners’ Rating Scales filled out by several informants (Teacher’s: CTRS, self-rated: CAARS [22, 23]). This approach was chosen to ensure robust categorization into ADHD and NAC groups, considering that a diagnostic interview with a clinician may in some cases underestimate ADHD symptomatology due to social-desirability bias. To be diagnosed with ADHD, participants were required to have a minimum of 6 symptoms of either inattention or hyperactivity/impulsivity (or 5 for participants ≥ 18 years), that arose before the age of 12, were present in ≥ 2 settings and led to impairment. Participants were categorized as NAC if they had ≤ 3 symptoms (or ≤ 2 for participants ≥ 18 years). The following exclusion criteria applied for both the ADHD and NAC groups: an IQ < 70, a diagnosis of autism, epilepsy, general learning difficulties, brain disorders or known genetic disorders (see [17] for a more detailed description of diagnostic and inclusion/exclusion procedures). Participants who neither met the criteria for the ADHD nor for the NAC group were classified as ‘subthreshold ADHD’ and were excluded from the current analyses.

#### Medication use

Medication history data were obtained from pharmacy records and a semi-structured interview to reconstruct lifetime history of stimulant medication use. Pharmacy records were used to encode medication history, unless unavailable, in which case the semi-structured interview was used to reconstruct lifetime history of stimulant medication use. Those ADHD participants that never received ADHD psychostimulants were categorized as ADHD-nostim, whereas ADHD participants with exposure to ADHD psychostimulants were categorized as ADHD-stim.

#### ADHD symptoms and comorbidities

The validated parent-rated Conners’ Rating Scales (CPRS R: L [24]) was used for symptom network analysis, as CPRS data was not used to establish ADHD diagnosis or determine NAC status. The CPRS consists of 80 behavioral statements, of which 18 describing ADHD symptoms that reflect ADHD symptoms in the DSM-IV. The 18 items used for symptom network estimation are shown in Table 1. Parents were instructed to indicate how often their child exhibits each of the behaviors listed on a 4-point Likert scale (0 = never, 1 = occasionally, 2 = often, 3 = very often). For those participants that were using stimulant medication, the parents were asked to rate the CPRS based on their child’s behavior as it would be without medication use. History of comorbid disorders was assessed using the K-SADS-PL [21].

### Data analysis

All analyses were performed in R (version 4.0.5) using the packages *mgm* (version 1.2–14 [25]), *qgraph* (version 1.9.2 [26]), *ggplot2* (version 3.4.0), and *mice* (version 3.14 [27]). For creation of the stimulant treatment trajectory groups, R version 4.1.2 and Matlab (version R2016a) were used. For descriptive analyses, a significance level of *α* = 0.05 was applied.

Since symptom network analysis does not allow for missing data, missing CPRS items for participants with incomplete CPRS data were imputed using multiple imputation. Participants with ≥ 50% missing CPRS data or incomplete medication use data were excluded from analysis.

#### Symptom network construction

To assess the association of stimulant medication with ADHD symptom interrelations, we compared symptom networks between NACs, ADHD-nostim and ADHD-stim participants using moderated network models. In this approach, a symptom network is estimated across all participants and a categorical moderator variable is included in the network to evaluate the networks for each moderator group [28]. Moreover, we performed additional network analyses as sensitivity analyses (see Sect. “Sensitivity analyses”).

**Table 1:** Attention-deficit/hyperactivity disorder (ADHD) symptoms assessed using the Conners’ parent rating scale (CPRS R: L). Overview of symptom labels used throughout this paper and the corresponding DSM-IV criteria for ADHD

Symptom label	DSM-IV criteria for ADHD
**Inattentive**	
Closeatt	Often fails to give close attention to details or makes careless mistakes in schoolwork, work, or other activities
Susatt	Often has difficulty sustaining attention in tasks or play activities
Listen	Often does not seem to listen when spoken to directly
Instruct	Often does not follow through on instructions and fails to finish schoolwork, chores, or duties in the workplace
Org	Often has difficulty organizing tasks and activities
Avoid	Often avoids, dislikes, or is reluctant to engage in tasks that require sustained mental effort
Lose	Often loses things necessary for tasks or activities
Distract	Is often easily distracted by extraneous stimuli
Forget	Is often forgetful in daily activities
**Hyperactive-impulsive**	
Fidget	Often fidgets with hands or feet or squirms in seat
Seat	Often leaves seat in classroom or in other situations in which remaining seated is expected
Run	Often runs about or climbs excessively in situations in which it is inappropriate
Quiet	Often has difficulty playing or engaging in leisure activities quietly
Motor	Is often “on the go” or often acts as if “driven by a motor”
Talk	Often talks excessively
Blurt	Often blurts out answers before questions have been completed
Turn	Often has difficulty awaiting turn
Interrupt	Often interrupts or intrudes on others

We estimated mixed graphical models (MGMs) to evaluate the networks of ADHD symptoms. In these networks, the nodes represent the 18 ADHD symptom scores assessed using the CPRS and the edges represent statistical associations between pairs of symptoms while controlling for all other variables in the network (i.e., conditional dependence relationships [29, 30]). Given our relatively low sample size compared to the number of included variables, we used LASSO regularization with the Extended Bayesian Information Criterion (EBIC) to remove spurious edges (γ = 0.5 [31, 32]). We included edges using the OR-rule and assessed the stability of the estimated moderation parameters by performing non-parametric block bootstrapping analysis (*n* = 500 bootstrap samples). Moderation effects that were present in 50–80% (moderate stability) or ≥ 80% (good stability) of the bootstrap samples were considered to have sufficient stability for interpretation.

#### Local network properties

To characterize the role of particular symptoms in the ADHD symptom networks, we computed two local metrics describing local network properties: node strength and a local clustering coefficient. These metrics were selected as they take edge weights into account [33, 34] and allow for two different ways to evaluate network properties. Node strength reflects the sum of the absolute weights of the edges connected to a node [35], whereas the local clustering coefficient reflects the proportion of edges present between a node’s neighbors in relation to all possible edges (calculation adapted for weighted networks, as developed by [36]). Finally, we compared local network properties of the NAC, ADHD-nostim and ADHD-stim groups by assessing how strongly correlated the local network metrics (node strength and local clustering coefficients) were between groups.

#### Visualization

We visualized the networks conditioned on the moderator groups, using the Fruchterman-Reingold algorithm, such that more strongly associated nodes are plotted more closely together [37]. To facilitate the comparison across groups, we fixed the layout and maximum edge weight across all visualizations. Edge thickness corresponded to the strength of associations between symptoms, with thicker edges representing stronger associations.

### Sensitivity analyses

#### Additional symptom network analyses

The main analysis considered the NAC, ADHD-nostim and ADHD-stim groups as completely separate groups, and did not consider that all participants in the ADHD-nostim and ADHD-stim groups had an ADHD diagnosis. To investigate a potential association of ADHD diagnosis with ADHD symptom interrelations, we conducted an additional network analysis comparing the NAC and total ADHD groups.

We conducted several sensitivity analyses in our study. First, we included age (continuous) and sex (0: female; 1: male) in the network model to account for their potential impact on the associations between ADHD symptoms, given the known sex differences and developmental changes in ADHD type [38, 39]. Additionally, we included study site (Amsterdam, Nijmegen) in the network model to account for its potential influence on the associations between ADHD symptoms. Furthermore, we repeated the main analysis including only participants with complete CPRS data (complete cases).

Finally, we conducted an additional network analysis comparing subgroups defined by their stimulant treatment trajectory (groups defined by the community detection algorithm described in Sect. “Stimulant treatment trajectories”).

#### Stimulant treatment trajectories

Considering medication use trajectories in ADHD are highly heterogeneous, the ADHD group was further divided into subgroups defined by their stimulant treatment trajectory using a community detection algorithm, as previously described [40, 41]. The algorithm classifies participants by maximizing similarities within groups as well as differences between groups, until separation of subgroups no longer improves with further iterations. The modularity (Q) reflects the separation of subgroups, with Q = 0 indicating no subgroups and Q = 1 indicating perfect segregation between subgroups. Lifetime stimulant trajectories (mg per day) were constructed for each participant from date of birth to date of questionnaire assessment, and averaged per month. We extracted age of medication treatment onset, stop age, and total dose from the raw treatment trajectories. Treatment duration, maximum daily dose, and variability (SD) of the dose were estimated from a generalized additive model (GAM [42, 43]) fitted to the raw trajectories. Next, these variables were entered into a Louvain community detection algorithm [44], in order to categorize participants into mutually exclusive communities (stimulant treatment trajectory subgroups).

## Results

### Participant characteristics

From the original sample (*n* = 1085), 494 participants were classified as NAC, and 432 as ADHD participants. A total of 864 participants (472 NAC, 392 ADHD) had complete CPRS data, and 24 participants had > 50% (1 NAC, 1 ADHD) or completely missing (11 NAC, 11 ADHD) CPRS data. For 38 participants with incomplete CPRS data (10 NAC, 28 ADHD; ≤4 missing items), missing CPRS scores were imputed using multiple imputation. Additionally, 25 participants (23 NAC, 2 ADHD) with incomplete stimulant medication history data and NACs with exposure to stimulant medication (*n* = 15) were excluded from analysis, resulting in a final sample of 862 participants (444 NAC, 70 ADHD-nostim, 348 ADHD-stim).

Table 2 shows the demographic information for the NAC and total ADHD groups, and separately for the ADHD-nostim and ADHD-stim groups. The ADHD-stim group had higher median ADHD sum scores compared with the ADHD-nostim group. Furthermore, the total ADHD group had a lower proportion of females compared with NACs, and the ADHD-stim group had a lower proportion of females compared with the ADHD-nostim group. In addition, the total ADHD group showed higher prevalence of comorbid disorders compared with the NACs. However, the ADHD-nostim and ADHD-stim groups did not differ in terms of comorbid disorders, suggesting that comorbid disorders were more common among participants with ADHD, regardless of the specific treatment they received. The duration of stimulant medication use in the ADHD-stim group ranged from 1 month to 10.3 years. One participant in the ADHD-nostim group may previously have used atomoxetine as treatment for ADHD for approximately one month.

**Table 2:** Participant characteristics. Data are presented as mean ± standard deviation, median (interquartile range) or *n* (%)

	NAC	ADHD (total)	Statistics^a^	ADHD-nostim	ADHD-stim	Statistics^a^
	*n* = 444	*n* = 418		*n* = 70	*n* = 348	
Age (years) * mean ± SD*	16.9 ± 3.9	16.5 ± 3.6	t(860) = 1.31, *P* = .19	17.3 ± 4.4	16.4 ± 3.4	t(86) = 1.72, *P* = .09
Sex * n (% female)*	245 (55.2%)	136 (32.5%)	Χ^2^(1) = 43.8, *P* < .001	34 (48.6%)	102 (29.3%)	Χ^2^(1) = 8.99, *P* = .003
IQ^b^ * mean ± SD*	103.7 ± 15.0	95.6 ± 16.5	t(780) = 7.20, *P* < .001	98.7 ± 16.5	95.0 ± 16.4	t(386) = 1.64, *P* = .10
ADHD sum score^c^ * median (IQR*)	3 (1–6)	24 (16–31)	W = 8896, *P* < .001	17 (9–25)	25 (17–32)	W = 7963, *P* < .001
Study site * n (% Amsterdam)*	269 (60.6%)	198 (47.4%)	Χ^2^(1) = 14.62, *P* < .001	55 (78.6%)	143 (41.1%)	Χ^2^(1) = 31.35, *P* < .001
Comorbid disorders^d^ * n (% present*)	15 (3.4%)	145 (34.7%)	Χ^2^(1) = 137.57, *P* < .001	22 (31.4%)	123 (35.3%)	Χ^2^(1) = 0.24, *P* = .62
CD	0 (0.0%)	25 (6.0%)	Χ^2^(1) = 25.27, *P* < .001	1 (1.4%)	24 (6.9%)	Χ^2^(1) = 2.20, *P* = .14
ODD	10 (2.3%)	133 (31.8%)	Χ^2^(1) = 133.89, *P* < .001	19 (27.1%)	114 (32.8%)	Χ^2^(1) = 0.61, *P* = .44
Anxiety disorder	3 (0.7%)	8 (1.9%)	Χ^2^(1) = 1.73, *P* = .19	3 (4.3%)	5 (1.4%)	Χ^2^(1) = 1.23, *P* = .27
Avoidant disorder/social phobia	1 (0.2%)	2 (0.5%)	Χ^2^(1) = 0.003, *P* = .96	0 (0.0%)	2 (0.6%)	Χ^2^(1) < 0.001, *P* > .99
Mood disorder	2 (0.5%)	11 (2.6%)	Χ^2^(1) = 5.50, *P* = .02	3 (4.3%)	8 (2.3%)	Χ^2^(1) = 0.29, *P* = .59

NAC = non-ADHD control, ADHD = attention-deficit/hyperactivity disorder, ADHD-nostim = stimulant treatment-naive ADHD participants, ADHD-stim = ADHD participants with exposure to stimulant medication, CD = conduct disorder, ODD = oppositional defiant disorder, IQ = intelligence quotient

^a^ Unpaired two-sample t-test, Chi-squared test or Mann Whitney U test

^b^ Estimated using the block design and vocabulary subtests of the Wechsler Intelligence Scales for Children and Adults (WISC-III / WAIS-III)

^c^ Assessed using the Conners’ Parent Rating Scale (CPRS-R: L)

^d^ Assessed using the (Kiddie - Schedule for Affective Disorders and Schizophrenia Present and Lifetime Version (K-SADS-PL)

### Symptom network analysis

#### Association of stimulant medication with ADHD symptom networks

**Fig. 1:**
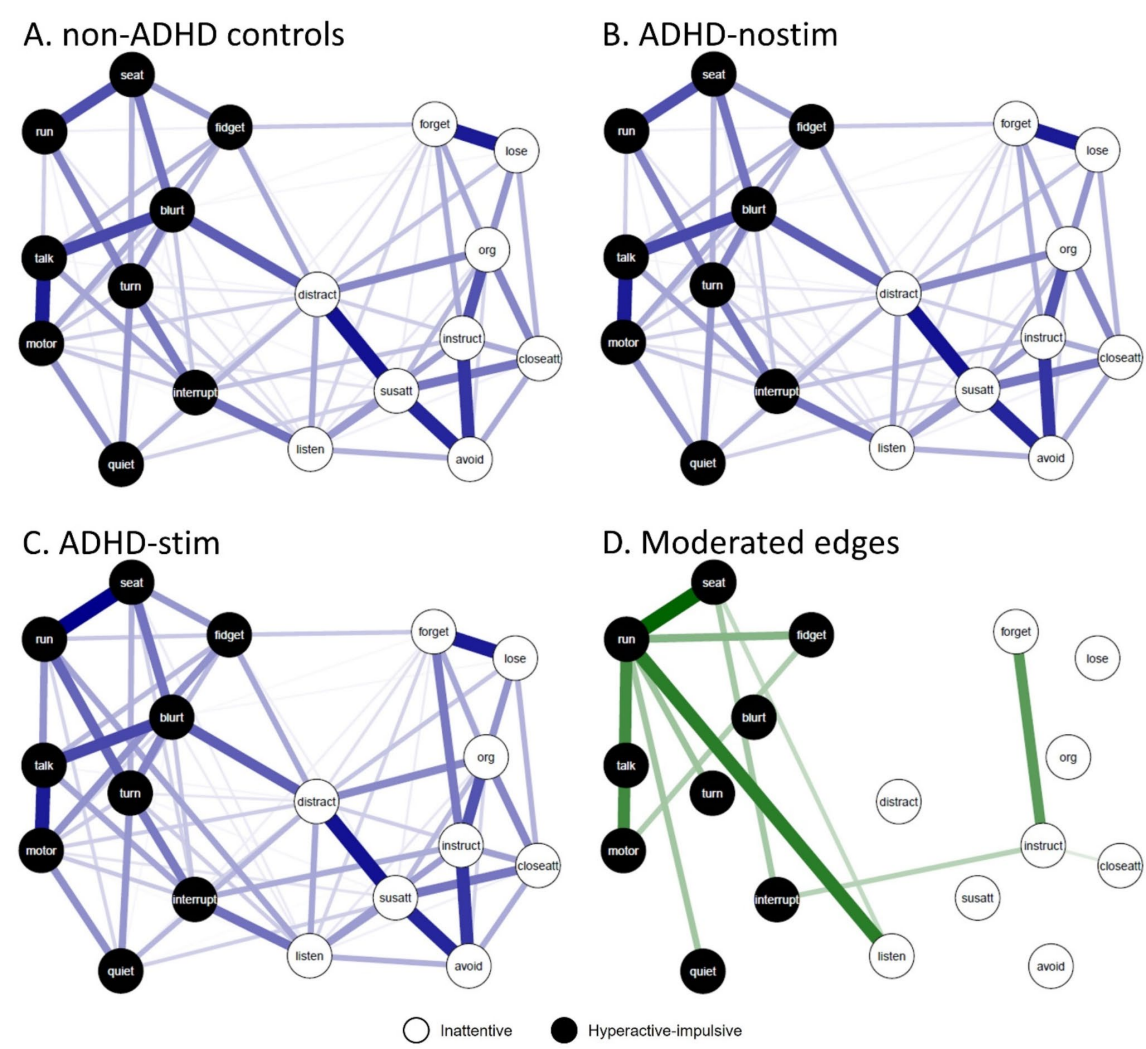
Symptom networks of stimulant-treated (ADHD-stim) and untreated (ADHD-nostim) individuals with attention-deficit/hyperactivity disorder (ADHD) and non-ADHD controls (NACs). (**A**) ADHD symptom network visualized for NACs. (**B**) ADHD symptom network visualized for ADHD-nostim participants. (**C**) ADHD symptom network visualized for ADHD-stim participants. (**D**) Difference in symptom networks of the ADHD-nostim group compared with the NAC and ADHD-stim groups, reflecting the moderation effects identified when comparing the NAC, ADHD-nostim, ADHD-stim networks. Edges represent the associations between symptoms while controlling for all other variables in the network and edge thickness represents correlation strength. In all three networks, all associations between symptoms were positive. All moderation effects were positive, indicating stronger associations between symptoms in the ADHD-stim network compared with the NAC and ADHD-nostim networks. The maximum edge weight was 0.29 for the NAC, ADHD-nostim and ADHD-stim networks, and 0.09 for the network of moderated edges

Symptom networks of the NAC, ADHD-nostim and ADHD-stim networks are visualized in Fig. 1A-C. For all networks, all edges were positive, indicating positive partial correlations between symptoms. Network analysis comparing NACs, ADHD-nostim and ADHD-stim participants revealed 12 moderation effects, of which 11 had sufficient stability for interpretation (4 good stability, 7 moderate stability; Fig. 1D; Table 3). Most of the identified stable moderation effects were on edges with hyperactive-impulsive symptoms, with 7 moderation effects on edges between hyperactive-impulsive symptoms, 3 moderation effects on edges between hyperactive-impulsive and inattentive symptoms, and one moderation effect on an edge between inattentive symptoms. Post hoc evaluation revealed that the strength of associations between symptoms was higher in the ADHD-stim network compared with the NAC and ADHD-nostim networks (Table 3), and that the moderated edges did not differ between the NAC and ADHD-nostim groups. In other words, the identified moderation effects reflected differences between the ADHD-stim group and the other two groups.

**Fig. 2:**
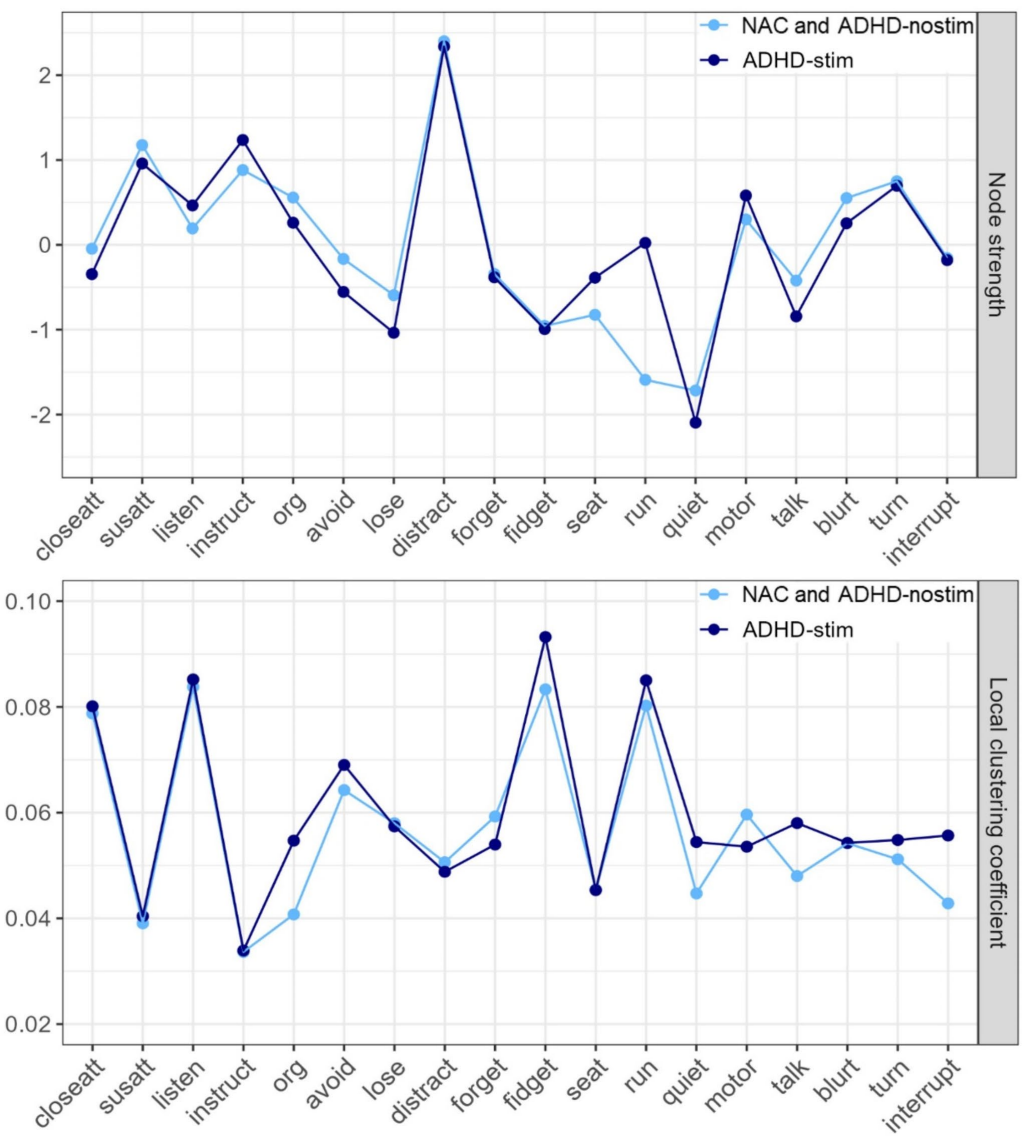
Local network properties of symptom networks in stimulant-treated (ADHD-stim) and untreated (ADHD-nostim) individuals with ADHD and non-ADHD controls (NACs). Standardized local network metrics (node strength and local clustering coefficients) describing local network properties, calculated from the NAC, ADHD-nostim and ADHD-stim networks. Note that the symptom networks and local network properties did not differ between the NAC and ADHD-nostim groups. The Pearson correlation coefficient between the local network properties of the ADHD-stim and NAC/ADHD-nostim groups was 0.88 for node strength and 0.93 for local clustering coefficients

Regarding local network properties (Fig. 2), mean ± standard deviation of the node strength was 0.81 ± 0.15 in the ADHD-stim network and 0.75 ± 0.16 in the NAC and ADHD-nostim networks. Furthermore, the mean ± standard deviation of the local clustering coefficients was 0.061 ± 0.016 in the ADHD-stim network and 0.057 ± 0.015 in the NAC and ADHD-nostim networks. Note that since the NAC and ADHD-nostim networks did not differ, their local network properties also did not differ. The Pearson correlation coefficient between the local network properties of the ADHD-stim and NAC/ADHD-nostim groups was 0.88 for node strength and 0.93 for local clustering coefficients. This suggests that all groups were similar in (local) network structure.

**Table 3:** Moderation effects identified in the moderated network analysis comparing stimulant-treated (ADHD-stim) and untreated (ADHD-nostim) individuals with attention-deficit/hyperactivity disorder (ADHD) and non-ADHD controls (NACs). The table shows the moderated edges, the proportion of bootstrap samples in which the moderation effect was present, and the edge weights of the moderated edges in the NAC, ADHD-nostim and ADHD-stim networks. Moderation effects that were present in 50–80% (moderate stability) or ≥ 80% (good stability, shown in bold) of the bootstrap samples were considered to have sufficient stability for interpretation. Note that the NAC and ADHD-nostim networks did not differ, and that therefore the differences in network structure were related to medication use in the ADHD group

Moderated edge	Proportion bootstrap samples^a^	Edge weight NACs	Edge weight ADHD-nostim	Edge weight ADHD-stim
***Run - motor***	**95%**	**0.056**	**0.056**	**0.123**
***Seat - run***	**91%**	**0.199**	**0.199**	**0.288**
***Listen - run***	**90%**	**0.028**	**0.028**	**0.102**
***Run - turn***	**80%**	**0.140**	**0.140**	**0.174**
*Fidget - run*	78%	0.020	0.020	0.062
*Instruct - interrupt*	69%	0.069	0.069	0.094
*Seat - interrupt*	67%	0.020	0.020	0.051
*Fidget - motor*	61%	0.095	0.095	0.123
*Listen - seat*	60%	0.000	0.000	0.021
*Instruct - forget*	59%	0.094	0.094	0.152
*Run - quiet*	58%	0.015	0.015	0.048
*Closeatt - instruct*	47%*			

NAC = non-ADHD controls, ADHD = attention-deficit/hyperactivity disorder, ADHD-nostim = stimulant treatment-naive ADHD participants, ADHD-stim = ADHD participants with exposure to stimulant medication

^a^ Proportion of bootstrap samples in which the moderation effect was identified

* Pairwise edges per network not shown, since these moderation effects had insufficient stability for reliable interpretation

#### Sensitivity analyses

##### Additional symptom network analyses

Network analysis comparing the NAC and total ADHD groups revealed 15 moderation effects, however stability analyses indicated that none of the moderation effects had sufficient stability for reliable interpretation of the moderated edges (Online Resource 1). This supports the findings of the main analysis that the identified moderation effects reflected differences in symptom networks between the ADHD-stim group and the other two groups. The sensitivity analyses with age, sex, and study site included in the network model yielded all moderation effects identified in the main analysis, as well as two additional stable moderation effects involving age and sex (Online Resource 2 and 3). Network analysis with complete cases only revealed 13 moderation effects, of which 11 were overlapping with the moderation effects identified in the main analysis (Online Resource 3). Of note, the two additional non-overlapping moderation effects identified when including complete cases only, had insufficient stability for reliable interpretation.

##### Stimulant medication use trajectories

The community detection algorithm yielded three subgroups (Q = 0.59) reflecting stimulant treatment trajectories (similar to [40, 41]): early-and-intense use (*n* = 153, 52%), late-and-moderate use (*n* = 140, 47%), and early-and-moderate use (*n* = 4, 1%). Due to insufficient sample size, the early-and-moderate group was excluded from analysis. Compared with the late-and-moderate group, the early-and-intense group was characterized by earlier medication treatment onset, higher total and maximum dose, higher variability of the dose, longer treatment duration, and earlier stop age (Online Resource 4–6). Network analysis comparing the early-and-intense and late-and-moderate subgroups revealed 2 moderation effects, however stability analysis indicated that neither moderation effects had sufficient stability for reliable interpretation of the moderated edges (Online Resource 7).

## Discussion

The present study aimed to investigate the association of stimulant medication with ADHD symptom networks. We identified multiple moderation effects, mostly involving hyperactive-impulsive symptoms, suggesting stronger associations between symptoms in stimulant-treated ADHD participants compared with untreated ADHD participants and NACs. This partially aligns with previous findings that hyperactive-impulsive symptoms had higher relative importance in symptom networks of ADHD participants [14]. In addition, local network properties were highly correlated across groups, suggesting that the identified differences are related to strength of symptom associations rather than (local) network structure. Findings were robust when taking into account age, sex and study site, and when including complete cases only. Moreover, we found no differences in symptom networks between the different stimulant treatment trajectory subgroups.

In contrast to our hypothesis, our findings provide no evidence for differences in symptom networks between stimulant treatment-naive participants with ADHD and NACs, and suggest that exposure to stimulant medication in the ADHD group was associated with stronger connections between symptoms. An explanation for our findings may be that untreated individuals with ADHD constitute a distinct subpopulation that presents with lower ADHD symptom severity and a symptom network similar to NACs, as substantiated by our findings of lower ADHD symptom severity in the ADHD-nostim group compared with the ADHD-stim group. Consequently, this particular subpopulation of untreated individuals with ADHD may be less reliant on pharmacotherapy. Longitudinal studies (in medication-naive participants) are required to disentangle whether the identified differences in symptom interrelations are a result of stimulant treatment or due to pre-existing differences between stimulant-treated and untreated individuals with ADHD. In the absence of such studies in ADHD, we propose two explanations for our findings based on studies investigating treatment effects on symptom networks in related fields. First, the heightened interconnectedness observed among hyperactive-impulsive symptoms may arise as a consequence of stimulant treatment. Previous studies have reported that symptom networks undergo changes following (psychological and/or medication) treatment in various neuropsychiatric populations [45–47]. Although different treatments may affect symptom networks differently [48], it is conceivable that stimulant medication may exert influence on specific associations between ADHD symptoms. A second, alternative explanation could be that the heightened interconnectedness observed in the symptom networks of stimulant-treated individuals with ADHD predates the initiation of stimulant treatment, implying that individuals with a more connected network of symptoms were more likely to be prescribed stimulants in the first place and/or are more responsive to stimulant treatment. In other words, the presence of a highly interconnected symptom network may correlate with greater impairment [30], subsequently prompting the administration of stimulant medication.

The findings of the present study provide a first insight into the association between stimulant medication and symptom networks in ADHD. This is of great significance, considering the widespread use of stimulants in the treatment of ADHD, as well as substantial interindividual variability in treatment response and tolerability of this medication [11, 49]. Currently, there is a scarcity of research exploring the causal effects of treatment on symptom networks in general (see [50] for a systematic review of network analysis in intervention studies), making further exploration of this matter crucial to enhance our understanding of the adaptability of symptom networks, specifically whether they reflect enduring traits or transient states. Notably, studies conducted in individuals with depression and schizophrenia found that treatment response was associated with the strength of associations between symptoms as well as symptom network changes compared with pre-treatment [45, 46]. Hence, future studies should focus on longitudinal evaluation of stimulant medication effects on ADHD symptom networks and discerning how these effects correspond to clinical outcomes. Furthermore, an outstanding question remains how symptom interrelations in ADHD relate to functional brain measures. Previous research focused mainly on connectivity at either a symptom level [14–16, 38, 51–54] or at a neural level [55–58]. Future studies will move towards an integrated network approach as previously proposed [59] to investigate the complex brain-behavior relationship in ADHD, as well as the influence of stimulant treatment thereon.

The findings of this study should be interpreted in light of some methodological considerations. First, the untreated ADHD group and the stimulant treatment trajectory subgroups had limited sample sizes, resulting in lower stability of the estimated symptom networks [60]. Nonetheless, robustness of our results was evaluated using sensitivity analyses, which revealed that the identified differences in the networks across groups were stable, and provided no evidence for network differences between participants with ADHD and NACs. Future research should make use of pooled datasets to benefit from larger sample sizes and explore potential ADHD subpopulations. Moreover, self-report and parent-report ratings of ADHD symptoms have previously been found to differ substantially [38, 61], although there is no conclusive evidence favoring the accuracy of self-report or parent-report ratings in adults [62, 63]. For reporter consistency and because the self-report CAARS was used for allocation into ADHD and NAC groups, we considered use of CPRS data for symptom network estimation most suitable. Still, given the subjective nature of the CPRS ratings, parents of stimulant-treated ADHD participants may vary in their interpretations of the instructions. They might assess their child’s behavior based on different timeframes – off-medication days, pre-treatment behavior, or a combination of both. This highlights the need for clear guidelines and detailed reporting on rating criteria in studies examining treatment impacts on behavior. Furthermore, ADHD symptom severity and presence of comorbid disorders differed across groups, although stimulant-treated and untreated participants with ADHD did not differ in terms of comorbid disorders. A previous found that the inattentive and hyperactive-impulsive symptom domains in ADHD were associated with other cognitive and comorbid factors [64], and the existing literature presents mixed findings regarding stimulant treatment response in ADHD in relation to co-occurring disorders [65–68]. This highlights the need for further exploration of the relation between symptom interactions, cognitive functioning and comorbidities in the context of stimulant treatment for ADHD. In addition, this study’s sample consisted of Dutch Caucasian participants, limiting the generalizability of our findings to the worldwide population. Finally, it is important to acknowledge that interpretation of our findings is limited by this study’s cross-sectional design. To make inferences about intra-individual medication effects on ADHD symptom interactions, longitudinal designs such as single-case experimental designs (SCEDs) are needed, in which individuals are followed across time while they receive stimulant medication [69].

In conclusion, the present study is the first to investigate the association of stimulant medication with ADHD symptom interrelations. Our findings suggest that stimulant-treated participants with ADHD showed stronger associations between symptoms compared with stimulant treatment-naive ADHD participants and NACs, and that the identified differences in symptom networks were related to strength of symptom associations, rather than (local) network structure. Whether the identified differences in symptom networks are a result of stimulant treatment or due to pre-existing differences between stimulant-treated and untreated individuals with ADHD, remains to be investigated. Future research should focus on longitudinal evaluation of stimulant medication effects on symptom networks in ADHD, as well as on integration of symptom-brain networks to gain insight in the relation between the neural underpinnings and symptom interrelations in ADHD.

## Electronic supplementary material

Below is the link to the electronic supplementary material.

Supplementary Material 1

## Data Availability

The data that support the findings of this study are not openly available due to reasons of sensitivity. The data and code are available from the corresponding author upon reasonable request and execution of a data use agreement. Data are located in controlled access data storage at the Donders Institute for Brain, Cognition and Behavior - Donders Centre for Cognitive Neuroimaging - Radboud University Medical Center.

## Abbreviations

ADHD: Attention-deficit/hyperactivity disorder
NAC: Non-ADHD controls

## Footnotes

1 Please note that neuropsychiatric models consider ADHD to be a disorder. However, there is a growing acknowledgement of ADHD as part of neurodiversity; conceptualising ADHD as an extreme manifestation of naturally existing neurodivergent ways of being, thinking and acting [1, 2].
