# Supplementary material for: Stimulant medication and symptom interrelations in children, adolescents and adults with attention-deficit/hyperactivity disorder
Source: Eur Child Adolesc Psychiatry. 2024 Nov 11;34(6):1917–29. doi: 10.1007/s00787-024-02610-8 (PMC12198274; doi:10.1007/s00787-024-02610-8)
Supplement: Supplementary file 1 — Supplementary Material 1 [file 787_2024_2610_MOESM1_ESM.pdf]

Stimulant Medication and Symptom Interrelations in Children, Adolescents and Adults with Attention-Deficit/Hyperactivity Disorder

Supplemental Material

European Child & Adolescent Psychiatry

Zarah van der Pal[1*], Hilde M. Geurts[2], Jonas M.B. Haslbeck[3,4], Alex van Keeken[1], Anne Marijn Bruijn[1], Linda Douw[5], Daan van Rooij[6], Barbara Franke[7], Jan Buitelaar[6], Nanda Lambregts-Rommelse[6], Catharina Hartman[8], Jaap Oosterlaan[9], Marjolein Luman[9], Liesbeth Reneman[1], Pieter J. Hoekstra[10], Tessa F. Blanken#[3], Anouk Schrantee#[1]

# Contributed equally

Affiliations

[1]Amsterdam University Medical Center location University of Amsterdam, Department of Radiology & Nuclear Medicine, Amsterdam, The Netherlands.

[2]Division of Brain & Cognition, Department of Psychology, University of Amsterdam, Amsterdam, The Netherlands.

[3]University of Amsterdam, Department of Psychological Methods, Amsterdam, The Netherlands.

[4]Maastricht University, Department of Clinical Psychological Science, Maastricht, The Netherlands.

[5]Amsterdam University Medical Center location Vrije Universiteit Amsterdam, Department of Anatomy & Neurosciences, Amsterdam, The Netherlands.

[6]Donders Institute for Brain, Cognition and Behavior - Donders Centre for Cognitive Neuroimaging - Radboud University Medical Center, Department of Cognitive Neuroscience, Nijmegen, The Netherlands.

[7]Radboud University Medical Center, Department of Human Genetics, Nijmegen, The Netherlands.

[8]University Medical Center Groningen, Department of Psychiatry, Groningen, The Netherlands.

[9]Vrije Universiteit, Department of Clinical Neuropsychology, Amsterdam, The Netherlands.

[10]University of Groningen-University Medical Center Groningen, Department of Child and Adolescent Psychiatry, Groningen, The Netherlands.

***Corresponding author:** Zarah van der Pal; email: z.vanderpal@amsterdamumc.nl

Online Resource 1 Moderation effects identified in the moderated network analysis with 'diagnostic group' (non-ADHD controls, ADHD) as a moderator. The table shows the moderated edges and the proportion of bootstrap samples in which the moderation effect was present. Note that all moderation effects were present in <50% of the bootstrap samples, indicating insufficient stability for interpretation

Moderated edge	Proportion bootstrap samples[a]
seat - run	33%
instruct - avoid	33%
closeatt - forget	16%
susatt - turn	6%
listen - seat	5%
seat - interrupt	3%
distract - forget	2%
closeatt - quiet	1%
listen - run	0%
run - motor	0%
run - turn	<0.5%
seat - blurt	<0.5%
fidget - run	<0.5%
distract - quiet	<0.5%
distract - blurt	<0.5%

[a] Proportion of bootstrap samples in which the moderation effect was identified.

Online Resource 2 Sensitivity analysis with age, sex and study site taken into account

Sensitivity analysis with age and sex included in the moderated network model revealed all moderation effects identified in the main analysis, as well as two additional stable moderation effects involving age and sex (Online Resource 3). Sensitivity analysis with study site included in the network model yielded all moderation effects identified in the main analysis (Online Resource 3).

Online Resource 3 Moderation effects identified in the main analysis and the sensitivity analyses with age and sex, study site, and complete cases only. The table shows the moderated edges and the proportion of bootstrap samples in which the moderation effect was present. Moderation effects that were present in 50-80% (moderate stability) or ≥80% (good stability, shown in bold) of the bootstrap samples were considered to have sufficient stability for interpretation

**Moderated edges**	**Proportion bootstrap samples**[a]			
	**Main analysis**	**With age + sex**	**With study site**	**Complete cases**
Identified in main analysis				
run* - *motor	**95%**	**96%**	**94%**	**82%**
seat* - *run	**91%**	**88%**	**93%**	**94%**
listen* - *run	**90%**	**91%**	**91%**	**85%**
run* - *turn	**80%**	76%	77%	71%
fidget - *run*	78%	74%	77%	79%
instruct - *interrupt*	69%	65%	65%	64%
seat - *interrupt*	67%	60%	63%	69%
fidget - *motor*	61%	60%	58%	49%*
listen - *seat*	60%	59%	55%	-
instruct - *forget*	59%	59%	61%	52%
run - *quiet*	58%	65%	61%	63%
closeatt - *instruct*	47%*	43%*	51%	45%*
Additional				
age - *sex*	-	**98%**	-	-
run - *age*	-	**91%**	-	-
motor - *sex*	-	49%*	-	-
talk - *sex*	-	47%*	-	-
instruct - *org*	-	-	-	41%*
distract - *quiet*	-	-	-	35%*

[a] Proportion of bootstrap samples in which the moderation effect was identified.

* Insufficient stability for reliable interpretation.

- Moderation effect not identified.

Online Resource 4 Stimulant treatment trajectories

Prior to implementation of the community detection algorithm, 19 participants were excluded due to too large gaps in medication use information that could not be estimated using the GAM. Moreover, participants that were categorised as stimulant treatment-naïve (*n*=102) were excluded from analysis. Of note, here a different approach was used to determine which participants were stimulant treatment-naive than in the main analysis. A final sample of 297 ADHD participants was entered into the community detection algorithm.

Online Resource 5 Stimulant medication use per stimulant treatment trajectory group. Data are presented as mean ± standard deviation or median (interquartile range)

**Characteristic**	**Early-and-intense** *n* = 153	**Late-and-moderate** *n* = 140	**Statistics**[a]	**Comparison**
Age of treatment onset (years, *mean* ± *SD*)	6.8 ± 1.4	10.8 ± 2.8	t(201)=-14.95, P<.001	LM > EI
Stop age (years, *mean* ± *SD*)	14.5 ± 3.2	15.8 ± 3.2	t(291)=-3.65, P<.001	LM > EI
Treatment duration (years, *mean* ± *SD*)	7.7 ± 3.1	5.0 ± 3.2	t(291)=7.17, P<.001	EI > LM
Variability (*median (IQR)*)	235.8 (132.7-447.4)	53.7 (11.5-117.9)	W=18101, P<.001	EI > LM
Lifetime cumulative dose (mg, *mean* ± *SD*)	17.5 ± 8.5	5.7 ± 4.2	t(226)=15.27, P<.001	EI > LM
Maximum dose (mg, *mean* ± *SD*)	47.6 ± 20.3	22.0 ± 14.4	t(274)=12.51, P<.001	EI > LM

EI = early-and-intense, LM = late-and-moderate.

[a] Two-sample t-test or Mann-Whitney U test.

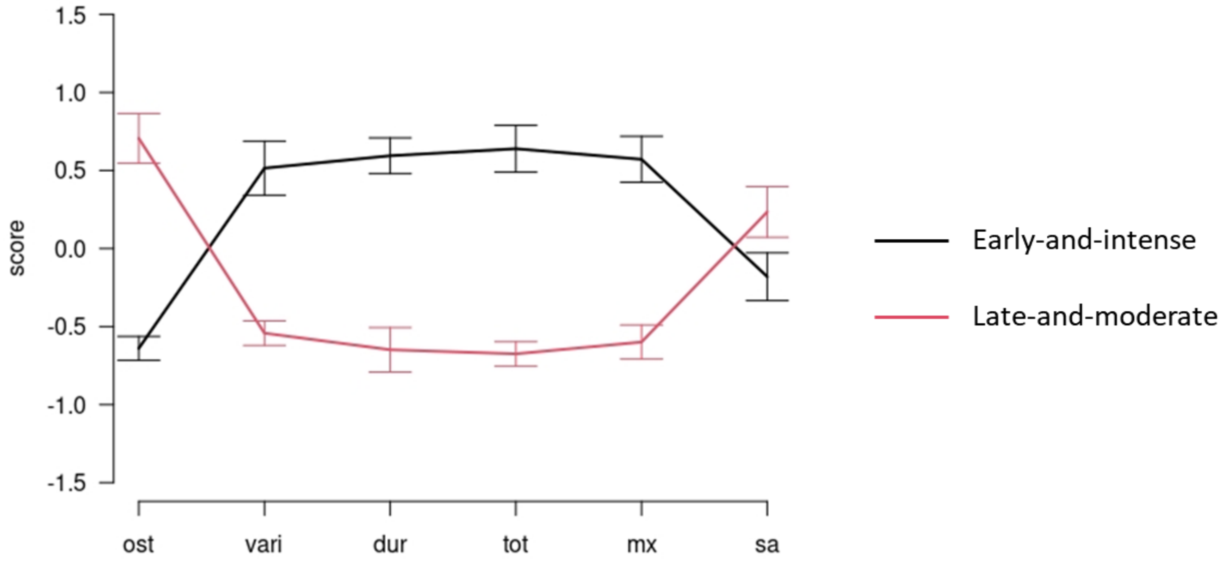

Online Resource 6 Stimulant treatment trajectory groups created using the community detection algorithm. ost = age of treatment onset, vari = variability, dur = treatment duration, tot = lifetime cumulative dose, mx = maximum daily dose, sa = stop age

Online Resource 7 Moderation effects identified in the moderated network analysis comparing the early-and-intense and late-and-moderate stimulant treatment trajectory groups. The table shows the moderated edges and the proportion of bootstrap samples in which the moderation effect was present. Note that all moderation effects were present in <50% of the bootstrap samples, indicating insufficient stability for interpretation

**Moderated edge**	**Proportion bootstrap samples**[a]
instruct - *org*	35%
distract - *talk*	6%

[a] Proportion of bootstrap samples in which the moderation effect was identified.
